# L'ostéome ostéoïde de l'extrémité inférieur du radius: à propos d'un cas, localisation rare et revue de la littérature

**DOI:** 10.11604/pamj.2016.24.46.5974

**Published:** 2016-05-11

**Authors:** Derfoufi Abdelhafid, Erraji Moncef, Kharraji Abdessamad, Abdeljaouad Najib, Yacoubi Hicham

**Affiliations:** 1 Unité de Chirurgie Orthopédique et Traumatologique, Centre Hospitalier Universitaire d'Oujda, Maroc

**Keywords:** Ostéome ostéoïde, extrémité inferieure du radius, exérèse, Osteoid osteoma, lower extremity of the radius, resection

## Abstract

L'ostéome ostéoïde est une tumeur osseuse bénigne, mais douloureuse et dont le traitement consiste en l'exérèse chirurgicale totale. Nous rapportons, dans ce travail le cas d'une jeune patiente présentant un ostéome ostéoïde de l'extrémité inférieur du radius.

## Abstract (en)

Osteoid osteoma is a benign but painful bone tumor whose treatment involves complete surgical resection. We report the case of a young patient with osteoid osteoma in the lower extremity of the radius.

## Introduction

L'ostéome ostéoïde est une tumeur osseuse bénigne douloureuse et pouvant se compliquer d'atteintes articulaires. Il affecte préférentiellement l'adolescent et le jeune adulte de sexe masculin. Il peut toucher tous les os, avec une prédominance pour les os longs. L'analyse anatomopathologique montre un nidus central hyper vascularisé, toujours inférieur à 2 cm, avec sclérose périphérique. Le traitement de référence est la chirurgie à ciel ouvert [1]. Nous rapportons, dans ce travail le cas d'une jeune patiente présentant un ostéome ostéoïde de l'extrémité inferieur du radius avec une revue de la littérature.

## Patient et observation

**Figure 1:**
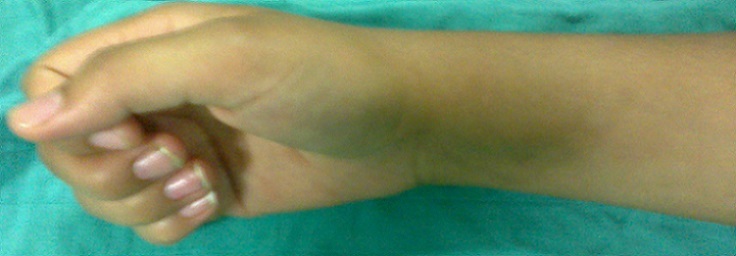
Aspect clinique

**Figure 2:**
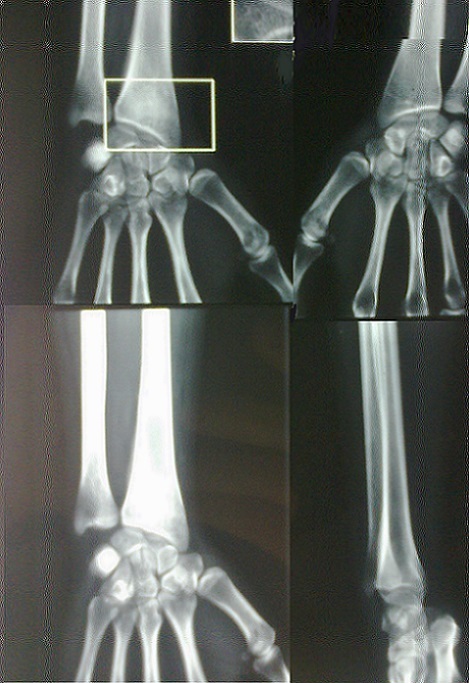
Image radiologique d'une lésion osteocondensante au niveau de l'extrémité inférieur du radius

**Figure 3:**
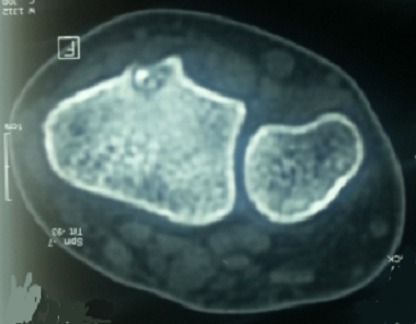
Image scanographique d'une lésion d'aspect compatible d'un ostéome ostéoïde

**Figure 4:**
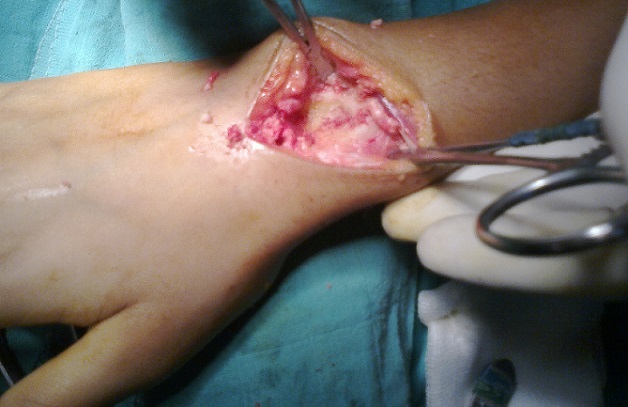
Exérèse chirurgical de la lésion

Mademoiselle R.H 20 ans sans antécédents pathologiques (pas de notion de traumatisme), se plaignant de douleurs au niveau de son poignet droit entrainant chez elle une impotence fonctionnelle ne lui permettant aucune activité depuis 1 an (Figure 1). A l'examen, douleur à la palpation en regard du tubercule de Lister. L'utilisation des anti-inflammatoires non stéroïdiens et des salicylées a permis une diminution de la douleur pendant les 2 premiers mois puis on a assisté à une recrudescence de la douleur malgré le traitement instauré. La douleur rapportée par la patiente n'a pas d'horaire particulier. L'imagerie radiologique à objectivé une image douteuse (ostéo-condensation) au niveau de l'extrémité inférieur du radius droit par comparaison au côté opposé (Figure 2). L'examen tomodensitométrique du poignet a objectivé au niveau de l'extrémité inférieur du radius une lésion d'aspect compatible d'un ostéome ostéoïde (Figure 3). Une intervention chirurgicale a permis de repérer la lésion suggestive de l'ostéome ostéoïde et on procéda à son exérèse (Figure 4). L'examen anatomopathologique confirma le diagnostic de l'ostéome ostéoïde. L’évolution post opératoire de la patiente était entièrement satisfaisante; on assiste à la disparition totale de tous les phénomènes douloureux ainsi qu'une reprise totale de toute activité au bout de trois mois.

## Discussion

L'ostéome ostéoïde est une tumeur osseuse primitive bénigne fréquente. Il représente 2 à 3% de l'ensemble des tumeurs osseuses et 10 à 20% de l'ensemble des tumeurs osseuses bénignes [2, 3]. L'extrémité inférieure du radius est une localisation très rarement rapportée. Il se situe préférentiellement au niveau des os longs [4] avec une prédilection pour les membres inférieurs [5], notamment le tibia et le fémur. Peu d'article de la littérature rapporte une telle localisation 1% des cas [1]. Les manifestations cliniques de l'ostéome ostéoïde sont le plus souvent faites de douleurs nocturnes, insomniantes, calmées par la prise de salicylés [5]. De ce fait, l'ostéome ostéoïde de l'extrémité inférieur du radius, malgré sa rareté, devrait toujours être considéré comme un diagnostic différentiel chez les jeunes patients se présentant avec une histoire douloureuse sans aucun antécédent de traumatisme [1]. le diagnostic clinique, la scintigraphie osseuse [2, 6], le scanner et dans certains cas l'IRM rendent le diagnostic quasiment certain avant la confirmation histologique [2, 3]. Néanmoins, ce diagnostic peut rencontrer de multiples difficultés, notamment devant des localisations inhabituelles notamment au niveau de l'extrémité inférieur du radius. En présence de toute atypie, une biopsie devra être pratiquée [5, 6]. Dans la littérature, pour le traitement de cette tumeur bénigne, bien qu'elle puisse évoluer spontanément après des années, plusieurs techniques sont utilisées: abord chirurgical avec l'exérèse osseuse en bloc [6] comme dans notre cas; résection percutanée scano-guidée [7]; alcoolisation percutanée: biopsie-résection percutanée par petites tréphines et sclérose par alcoolisation et destruction complète de la lésion [8].

## Conclusion

La localisation de l'ostéome ostéoïde au niveau de l'extrémité inférieur du radius est rare. En cas de doute diagnostic, la tomodensitométrie représente l'examen le plus spécifique permettant le diagnostic positif. L'exérèse chirurgicale complète de la lésion permet le plus souvent la guérison totale et évite les récidives. Elle peut être obtenue par chirurgie classique à ciel ouvert ou par techniques plus modernes mini-invasives: résection percutanée scano-guidé [7].
